# Bacterial cold-water disease in ayu (*Plecoglossus altivelis altivelis*) inhabiting rivers in Japan

**DOI:** 10.3389/fcimb.2022.1073966

**Published:** 2022-12-15

**Authors:** Masayuki Imajoh

**Affiliations:** Laboratory of Fish Disease, Aquaculture Course, Department of Marine Resource Science, Faculty of Agriculture and Marine Science, Kochi University, Nankoku, Kochi, Japan

**Keywords:** *Flavobacterium psychrophilum*, bacterial cold-water disease, ayu, river, Japan

## 1 Introduction

Ayu or sweetfish, *Plecoglossus altivelis altivelis*, is commercially important to inland fisheries in Japan and popular as a summer delicacy owing to its unusually sweet flavor. Ayu is also a popular recreational fishing species, especially for anglers using the Japanese fishing method “tomozuri.”

Bacterial cold-water disease (BCWD) was first recorded in an ayu farm in the Tokushima Prefecture in 1987 (Wakabayashi et al., 1994; Inouye, 2000). The cause of this occurrence was believed to be the introduction of ayu from Lake Biwa because BCWD was detected only a few days after landlocked ayu stock from Lake Biwa was transported to the farm (Wakabayashi, 2009). In 1993, BCWD spread to ayu populations in Gonokawa River in Hiroshima Prefecture (Iida and Mizokami, 1996). Subsequently, BCWD epizootics in ayu populations were reported in most rivers in Japan, and a close relationship was found to exist between the occurrence of BCWD and the release and use as a decoy of landlocked ayu stocks from Lake Biwa (Inouye, 2000; Taniguchi, 2002; Imura, 2003). According to the Japanese government statistics site e-Stat (https://www.e-stat.go.jp/en), the commercial catch of ayu in 2020 has decreased by 88.3% compared with the maximum catch of 16,414 tons in 1991. In the manuscript, the current knowledge of BCWD in ayu, which inhabits Japanese rivers, was compiled.

## 2 Types of native and stock ayu

Two forms of native ayu exist, amphidromous and landlocked ayu, with an assumed genetic distance and divergence time of approximately 100,000 years (Nishida, 1985). Amphidromous ayu is widely distributed in the Japanese Archipelago (Iguchi and Nishida, 2002) and has an annual life cycle as follows (Nishida, 1986): in autumn, mature ayu spawn and die after reproduction in the lower reaches of rivers; subsequently, newborn larvae hatch, flow to the sea, metamorphose into juveniles, and overwinter in habitats including estuarine and coastal environments (Murase et al., 2020); in spring, the juveniles migrate back into rivers and drift upstream where they grow during summer; when sexually mature, they drift downstream to the lower reaches of the river for spawning. In contrast, landlocked ayu is restricted to freshwater lakes. The largest landlocked population inhabits Lake Biwa, and the larvae and juveniles overwinter in the offshore water of the lake (Nishida, 1986).

Three types of stocking ayu exist: hatchery-born amphidromous, wild-born, and domesticated stocks. Most wild-born stocks comprise landlocked ayu caught in Lake Biwa; indeed, landlocked stock has been translocated into amphidromous populations in rivers throughout Japan for many years. Three concerns have been raised around the use of landlocked stock in relation to the conservation of natural amphidromous populations (Takamura, 2009; Kitada, 2022). First, the translocated landlocked stock poses a risk of interbreeding with wild amphidromous populations. Second, the landlocked stock cannot contribute successfully to the reproduction of the next generation. Third, the landlocked stock in Lake Biwa is infected with *Flavobacterium psychrophilum*. Amphidromous stock is genetically more resistant to BCWD than landlocked and domestic stocks (Nagai et al., 2004; Nagai and Sakamoto, 2006). For this reason, hatchery-born amphidromous stock is now produced and released into rivers in large quantities that surpass those of landlocked stock (Kitada, 2022).

The early release of *F*. *psychrophilum*-free amphidromous stock is recommended as a strategy for preventing the occurrence of BCWD in river-based ayu populations (Hara et al., 2007; Hara et al., 2008). The active cooperation of recreational anglers is also required to prevent BCWD, and the following recommendations have been proposed: voluntary restraints on moving ayu between rivers; cleaning fishing gears, including disinfection with alcohol and chlorine and drying in the sun or at a high temperature; and using different gears in different rivers (Katahira et al., 2019).

## 3 Genotype variation of *F*. *psychrophilum* isolates from ayu

Most *F*. *psychrophilum* isolates from ayu in Japan are serotype O-2; therefore, the serotyping approach is useful to determine host specificity (Wakabayashi et al., 1994; Iida and Mizokami, 1996; Izumi and Wakabayashi, 1999; Izumi et al., 2003b). Various genotyping techniques have been used to characterize their variations as follows: plasmid profiling assay (Izumi, 2004; Kim et al., 2010), pulsed-field gel electrophoresis assay (Arai et al., 2007), multiplex PCR–restriction fragment length polymorphism (PCR–RFLP) assay (Izumi et al., 2003a; Izumi et al., 2007; Izumi et al., 2019), on/off switch assay (Fujiwara-Nagata et al., 2012), and multilocus sequence typing (MLST) analysis (Fujiwara-Nagata et al., 2013) (**Table 1**).

**Table 1:** Summary of the studies investigating the distribution of *F*. *psychrophilum* in ayu populations in Japanese rivers and Lake Biwa.

Sampling river(s) or lake	Sampling month(s)	Catching method(s)	Health status	Prevalence of *F*. *psychrophilum* infection (detection method)	Serotyping or genotyping of *F*. *psychrophilum*(typing method(s))	Reference(s)
Hiroshima Prefecture						
Gonokawa River	Mid July to early November	Angling and gill nets	Healthy and diseased individuals	No examination	O-2 serotype (microtiter assay)	Iida and Mizokami (1996)*
**Okayama Prefecture**						
Kagami River	June and July	Not described	Dead individuals	0%–100% (Bacterial isolation)	No examination	Ueki et al. (1998)
	Late April to early July	Not described	Dead individuals	0%–100% (Bacterial isolation)	No examination	Ueki and Masunari (2000)
Niigata Prefecture						
Umikawa River	May to December	Casting nets and electrofishing	Healthy and diseased individuals	0%–51.3% and 0%–100% (IFAT** and PCR*** assays)	No examination	Amita et al. (2000)
Gunma Prefecture						
Tone River	May and June	Not described	Diseased and dead individuals	15%–88% (PCR assay)	No examination	Arai et al. (2004)
Agatsuma River, Karasu River, Kanna River, Tone River, and Watarase River	March to November	Not described	Diseased and dead individuals	No examination	A/S/XII-1, B/S/XII-2, A/S/XVI, A/S/XVII-1, A/S/XVII-2, A/S/XVII-3b, A/S/XVII-3c, and A/S/XVII-4 genotypes (both multiplex PCR-RFLP and PFGE assays)	Arai et al. (2007)
Hyogo Prefecture						
Makiyama River	May and June	Casting nets and gill nets	Healthy and diseased individuals	No examination	A/R and A/S genotypes (multiplex PCR-RFLP assay)	Tabata (2004)
Toyama Prefecture						
Sho River	Mid April	Electrofishing	Overwintering healthy individuals	39.1% (Bacterial isolation)	A/R/QR and A/S/QR genotypes (multiplex PCR-RFLP assay)	Miyazaki (2008)
Wakayama Prefecture						
Arida River	April to October	Casting nets and gill nets	Not described	0%–56.3% (PCR assay)	A/R, A/S, and B/S genotypes (multiplex PCR-RFLP assay)	Fujii et al. (2009)
Miyagi Prefecture						
Hirose River	May to November	Casting nets and angling	Healthy and diseased individuals	0%–100% (IFAT assay)	A/R, A/S, and B/S genotypes (multiplex PCR-RFLP assay)	Kumagai et al. (2010)
Isatomae, Mitobe, Aikawasawa, and Ohara Rivers	October	Casting nets and tangle nets	Healthy and diseased individuals	5%–95% (IFAT assay)	A/R, A/S, and B/S genotypes (multiplex PCR-RFLP assay)	Kumagai et al. (2011)
Tokyo						
Tama River	May to November	Handling nets and tomozuri angling	Healthy and diseased individuals	0%–97.7% (PCR assay)	A genotype (single PCR-RFLP assay)	Takeuchi et al. (2016)
Shiga Prefecture						
Lake Biwa and the inflowing rivers	November to August of the following year	Set nets, gill nets, offshore scoop nets, and fishing weir	Healthy and diseased individuals	0%–32.4% (PCR assay)	No examination	Yamamoto et al. (2015)
	June, September, and December	Electrofishing	Healthy and diseased individuals	0%–100% (PCR assay)	G-C and A-C genotypes (on/off switch assay)	Fujiwara-Nagata et al. (2019)
Kochi Prefecture						
Kagami River	October	Tomozuri angling	Healthy individual	No examination	A/G-C genotype (both single PCR-RFLP and on/off switch assays)	Shimizu et al. (2016)****;
	March to October	Casting nets, fly-fishing, and tomozuri angling	Healthy, diseased, and dead individuals	0%–100% (qPCR assay)	A/G-C/4.1-kbp plasmid, A/G-C/none, and B/A-C/2.4-kbp genotypes (both plasmid profiling and single PCR-RFLP assays)	Imajoh et al. (2017) ****;
	May and June	Tomozuri angling	Diseased individuals	No examination	ST45 and ST52 genotypes (MLST analysis)	Yamashita et al. (2019a)****
Monobe River	November and December	Casting nets	Healthy and dead individuals	73.1%–100% (qPCR assay)	No examination	Imajoh et al. (2021)****
Nahari River	April	Handling nets	Diseased individuals	No examination	ST45 and ST52 genotypes (MLST analysis)	Yamashita et al. (2019b)****
Shimanto River	November	Casting nets	Diseased individual	No examination	A/G-C/4.1-kbp plasmid genotype (plasmid profiling, single PCR-RFLP, and on/off switch assays)	Imajoh et al. (2017)****
	October and November	Casting nets	Healthy and dead individuals	77.8%–100% (5.2% in late October and 7.1% in early November) (qPCR assay)	A/G-C, A/A-C, A/A-T, and B/A-C genotypes (both single PCR-RFLP and on/off switch assays)	Imajoh et al. (2020)****
Aki River, Shimanto River, Matsuda River, Nahari River, Kagami River, Monobe River, None River, Niyodo River, Yasuda River, and Shinjo River	April to December	Not described	Diseased and dead individuals	No examination	A/G-C/R/ST45, A/G-C/S/ST52, A/G-C/S/ST65, A/A-C/R/ST45, A/A-C/R/unidentified ST, and B/A-C/S/unidentified ST genotypes (both multiplex PCR-RFLP assay and MLST analysis)	Urabe et al. (2021)
Not described	Not described	Not described	Not described	No examination	O-2 and untypable serotypes (microtiter assay)	Izumi and Wakabayashi (1999b)
Not described	Not described	Not described	Not described	No examination	O-1, O-2, O-2/4, O-4, and untypable serotypes (microtiter assay)	Izumi et al. (2003b)
Not described	Not described	Not described	Not described	No examination	A/R/QR/C, A/R/QS/C, A/S/QR/C, A/S/QR/D, A/S/QS/C, B/R/QS/C, B/S/QR/C, B/S/QR/D, B/S/QS/C, B/S/QS/D genotypes (multiplex PCR-RFLP assay)	Izumi et al. (2019)

*First report of the occurrence of BCWD in rivers. **Immunofluorescence antibody test. ***Polymerase chain reaction. ****Studies conducted by the author of this manuscript.

The single PCR–RFLP assay targets peptidyl-prolyl cis-trans isomerase C (*PPIC*) and divides the isolates into two genotypes: A and B types (Yoshiura et al., 2006). All A-type isolates are regarded as specific pathogens of ayu (Tabata, 2004), with only one B-type isolate causing BCWD in ayu in a bath infection challenge (Miwa and Nakayasu, 2005). Recently, Izumi et al. (2019) proposed the multiplex PCR–RFLP assay targeting the *PPIC*, DNA gyrase (*gyrA* and *gyrB*), and topoisomerase IV (*parE*) genes to improve genotyping performance and potentially allow the classification of 16 genotypes.

MLST provides more detailed genotyping data than the PCR–RFLP assay. MLST analysis based on seven housekeeping genes revealed that the CC-ST48, CC-ST52, and CC-ST56 lineages infect ayu in Japan and are important for the treatment and prevention of BCWD in ayu (Fujiwara-Nagata et al., 2013).

The on/off switch assay identifies two single nucleotide polymorphisms of *gyrA* and divides isolates into four genotypes: the G-C type isolated from ayu, the A-T type isolated from salmonid fish, and the G-T and A-C types isolated from several species including ayu (Fujiwara-Nagata et al., 2012). This assay assesses the potential pathogenicity of *F*. *psychrophilum* isolates to ayu: the G-C type shows strong pathogenicity, the A-T and G-T types show no pathogenicity, and the A-C type shows at most weak pathogenicity. Fujiwara-Nagata et al. (2019) determined the seasonal changes of the four genotypes in various samples collected from the lower basin of a river flowing into Lake Biwa, reporting that most of the isolates were the G-C type in September when ayu gathered in the lower basin for spawning and that the A-T type was only detected in December when Biwa trout (*Oncorhynchus masou rhodurus*) were present in the lower basin for spawning.

## 4 Seasonal distribution of *F*. *psychrophilum* in ayu

Ayu have been sampled to determine the distribution of *F*. *psychrophilum* in ayu populations in Japanese rivers and Lake Biwa (**Table 1**). Kochi Prefecture (**Table 1**) is located on the south coast of Shikoku Island in Japan; ayu inhabit the clear rivers of the prefecture, including the Kagami River, the Shimanto River, the Monobe River, and the Nahari River, although the commercial ayu catch has halved since 1993 due to the occurrence of BCWD (Taniguchi, 2002).

The Kagami River, located in central Kochi Prefecture, is 31 km long with a drainage basin area of 170 km^2^. The Kagami Dam in the middle of the river divides it into two streams, preventing ayu from drifting upstream or downstream. Thus, hatchery-born amphidromous stock is released in both the upper and lower reaches relative to the dam to enhance the natural ayu stocks. As reported by the Ministry of Agriculture, Forestry, and Fisheries, BCWD outbreaks most frequently occur from May to July at water temperatures of 14°C–21°C (http://www.maff.go.jp/j/syouan/suisan/suisan_yobo/ayu_reisui/). In late June 2014, there was a mass die-off of ayu in the upper reaches. Imajoh et al. (2017) inferred that the die-off was attributed to BCWD because they successfully isolated *F*. *psychrophilum* from all the collected dead individuals, and almost all isolates were identified as A/G-C types. Urabe et al. (2021) subsequently genotyped more isolates from more rivers in Kochi Prefecture using an on/off switch assay, a PCR–RFLP assay, and MLST analysis, finding that most isolates were the A/G-C/S/ST52 types. Therefore, this genotype is likely the main cause of BCWD in ayu in Kochi Prefecture’s rivers.

The Shimanto River, located in western Kochi Prefecture, is 196 km long with a drainage basin area of 2,186 km^2^. The Monobe River, located in central Kochi Prefecture, is 71 km long with a drainage basin area of 508 km^2^. The two rivers are considered Class A rivers, which are assigned by the government of Japan as important for the conservation of national land or for the national economy, and are famous for ayu, especially the Shimanto River, which possesses an abundance of native amphidromous ayu resources (Azuma et al., 2020). Many mature ayu drift down near the mouth of the river to lay their eggs in autumn, after which they die. Imajoh et al. (2020); Imajoh et al. (2021) collected 248 mature and 369 dead individuals at several times during the spawning season in the spawning grounds of the two rivers and used quantitative PCR to determine the prevalence of *F*. *psychrophilum* infection, which was very high at 73%–100% in the mature individuals, excluding late October and early November in the Shimanto River, and 100% in the dead individuals. Interestingly, many *F*. *psychrophilum–*infected dead individuals were prespawning fish. Sexual maturation is thought to decrease the resistance of ayu to *F*. *psychrophilum* infection as well as causing changes to nonspecific immune responses and lymphocytopenia (Minami et al., 2018; Kawashima et al., 2021). Therefore, acute *F*. *psychrophilum* infection likely occurs among mature ayu gathering on the spawning ground, resulting in septicemia due to the onset of BCWD.

Several catching methods for ayu sampling are shown in **Table 1**, and these methods differ according to the specific situation. There is a concern that some methods, especially tomozuri angling, may skew the catch toward healthy rather than diseased ayu because diseased fish exhibit lower physiological activity than healthy fish. Environmental DNA (eDNA) analysis enables year-round monitoring of ayu in rivers and Lake Biwa (Kono et al., 2017; Inui et al., 2018; Inui et al., 2019; Haga et al., 2020; Inui et al., 2020; Inui et al., 2021; Tsuji et al., 2022). Recently, the combined use of ayu and *F*. *psychrophilum* in eDNA analysis has attracted attention owing to its potential utility for predicting the occurrence of BCWD in rivers (Tenma et al., 2021). According to the studies of Strepparava et al. (2014) and Nguyen et al. (2018); Imajoh et al. (2020); Imajoh et al. (2021) selected the single copy gene β′ DNA-dependent RNA polymerase to detect *F*. *psychrophilum* in the water and conduct eDNA analysis in the Shimanto River and Monobe River, finding that both the eDNA concentrations of ayu and *F*. *psychrophilum* reached maximum levels in the river water of the spawning ground during the spawning season among the seasonal–annual distribution, likely reflecting the high prevalence of *F*. *psychrophilum* infection in mature and dead ayu at the spawning grounds.

## 5 Further perspectives: the necessity of assessing *F*. *psychrophilum* infection in spawning ayu

The findings presented in this manuscript indicate that *F*. *psychrophilum* infection can spread widely and rapidly in spawning ayu in rivers. Thus, it is necessary to estimate the extent to which spawning ayu are lost because of *F*. *psychrophilum* infection during the spawning season. It is also possible that *F*. *psychrophilum* released from spawning ayu could survive over winter and represent the preliminary infection source in the next year. This possibility is supported by a case report in the Nahari River (Yamashita et al., 2019b), which is located in the eastern Kochi Prefecture, is 61 km long, and has a drainage basin area of 311 km^2^. In April 2018, BCWD caused a mass die-off of juvenile ayu beginning to drift upstream near the river mouth, which received attention for being the first such occurrence during this month in Kochi Prefecture. Importantly, *F*. *psychrophilum* infection is considered not to have been introduced into the river from an outside source because (1) ayu stocks were not released and (2) no anglers fished on the river because of a fishing ban. Yamashita et al. (2019b) isolated six *F*. *psychrophilum* isolates from the dead individuals, determined their draft genome sequences, and examined their genotypes, which were in agreement with the genotypic results of Urabe et al. (2021). The obtained draft genome data will provide insights into the survival of *F*. *psychrophilum* over winter in the Nahari River and possible reinfection of the ayu population in the next spring.

## Author contributions

All authors contributed to the article and approved the submitted version.
